# An exploratory assessment of human and animal health concerns of smallholder farmers in rural communities of Chimborazo, Ecuador

**DOI:** 10.7717/peerj.12208

**Published:** 2022-01-17

**Authors:** Tamara L. Chavez-Lindell, Ana L. Moncayo, María Fernanda Vinueza Veloz, Agricola Odoi

**Affiliations:** 1 Biomedical and Diagnostic Sciences, University of Tennessee, Knoxville, TN, United States of America; 2 Centro de Investigación para la Salud en América Latina, (CISeAL), Escuela de Ciencias Biológicas, Facultad de Ciencias Exactas y Naturales, Pontificia Universidad Católica del Ecuador, Quito, Ecuador; 3 Facultad de Salud Pública, Escuela Superior Politécnica de Chimborazo, Riobamba, Chimborazo, Ecuador

**Keywords:** Health concerns, Ecuador, Smallholder farmers, Global health, Epidemiology, Smallholding, Pediatric diarrhea, Gastrointestinal illness, Sanitation, Firth logistic models

## Abstract

**Background:**

Livestock play important economic and cultural roles in smallholder communities of Ecuador, yet they also serve as potential sources of zoonotic infections. Understanding the animal and human health concerns of smallholder farmers is important in guiding strategies for improvement of the health and livelihoods of these resource-poor farmers. Therefore, the objectives of this study were to: (a) assess the health concerns of smallholder farmers; (b) explore animal and waste management practices; and (c) identify predictors of pediatric and livestock diarrhea on smallholder farms in Ecuador.

**Methods:**

This is a cross-sectional survey of 58 smallholder farmers in three communities of Chimborazo province, Ecuador. Data were collected on household demographics, smallholding characteristics, type of animals owned, human-animal interactions, health concerns, and 30-day occurrence of human as well as animal diarrhea. Summary statistics were computed and logistic models used to investigate predictors of pediatric and animal diarrhea.

**Results:**

All respondents reported keeping animals. Animals kept included cattle, pigs, poultry, dogs, guinea pigs, cats, sheep, horses, rabbits, donkeys, or other livestock. More than half of the respondents named diseases as their greatest personal (55.2%) or family (58.6%) health concern, while an even greater percentage (60.3%) reported physiological stress as the primary health concern for their animals. Occurrence of diarrhea in the 30 days prior to the study was reported by 12.1% of the respondents. Additionally, 15.2% and 55.2% of the households reported diarrhea among children and animals, respectively. The majority (65.5%) of the households had toilets, while the remainder had either latrines (27.6%) or no sanitation facilities (6.9%). However, only 9.1% of the smallholdings had either a toilet (3.6%) or a latrine (5.5%) onsite and yet the farmers tended to spend most of the day at the smallholdings. Potential exposures to gastrointestinal pathogens included food- or water-borne sources (93.5% of children; 91.4% of adults) and blood-borne or fecal sources (80.4% of children; 100% of adults). Although 98.3% of the respondents kept cattle, only 27.6% had animal enclosures and even fewer (15.5%) had animal waste management plans. The odds of animal diarrhea were significantly higher (Odds Ratio [OR] = 8.7; 95% Confidence Interval [1.0–75.0]; *p* = .049) among households that had animal waste management plans compared to those that did not. None of the variables investigated were significant predictors of pediatric diarrhea.

**Conclusions:**

Ongoing surveillance is needed to develop estimates of diarrhea incidence among smallholder families and their livestock. The impact of different animal management strategies on the potential pathogen exposure of smallholders warrants further investigations. Improving sanitation infrastructure and animal waste management strategies is recommended.

## Introduction

Domestic livestock are important sources of income in many smallholder communities of Ecuador, where, in addition to their economic role, they play vital cultural functions (Contreras Hernández, 1985). However, livestock also serve as sources of infections for a number of zoonotic diseases. Numerous causative agents of human gastrointestinal infections are zoonotic, (Olivier, Jayarao & Almeida, 2005; Zambrano et al., 2014) including various viruses, bacteria, and parasites that are common in Ecuador (Jacobsen et al., 2007; Praet et al., 2010; Atherton et al., 2013; Vasco et al., 2014; Carbonero et al., 2015; Gestal et al., 2015; Graham, Vasco & Trueba, 2016; Vasco, Graham & Trueba, 2016). Prevalence of pediatric diarrheal illness is estimated to be high nationwide, affecting a reported 17.0% of children under age 5 in a 2014 period prevalence survey (Instituto Nacional de Estadistica y Censos, 2015a). In the province of Chimborazo, the same survey identified pediatric diarrhea prevalence of 16.1% among children under 5 years of age (Instituto Nacional de Estadistica y Censos et al., 2015). County-level and parish-level data on pediatric diarrhea and associated outcomes are limited. However, the incidence of gastrointestinal infections in rural areas, where children regularly interact with livestock, would be expected to be higher than in urban areas where children have little to no interaction with farm animals and, hence, lower risk of cross-transmission of gastrointestinal pathogens between livestock and children. Indeed, the reported period prevalence of diarrhea among children under 5 years in the 2014 national living conditions survey was 25% higher in rural compared to urban residents (19.5% and 15.6%, respectively) (Instituto Nacional de Estadistica y Censos et al., 2015).

Pediatric diarrhea can have immense long-term impact, with outcomes of varying severity including malnutrition, malabsorption syndromes, failure to thrive, stunting, chronic bowel inflammation associated with ulcerative colitis, loss of educational attainment or productivity, dehydration, and death (Kotloff et al., 2013; Kotloff, 2017). Diarrhea and nutritional deficiencies are the principal drivers of underweight status in childhood, which in turn causes an estimated 1% of all disability-adjusted life years in the Ecuadorian population (Institute for Health Metrics and Evaluation, 2010). Despite the high burden of infectious diseases, national reporting of specific gastrointestinal infections is sporadic, given limited diagnostic testing capacity. Consequently, reporting categories largely reflect syndrome- or symptom-based functional diagnoses, including “diarrhea and infectious gastroenteritis” (the third most common cause of illness, with reported annual incidence in 2014 of 19/10,000 population (Instituto Nacional de Estadistica y Censos, 2014b)) and “water-related illnesses and conditions” (total reported annual incidence in 2014 of 25/10,000 population (Instituto Nacional de Estadistica y Censos, 2014b)). Although such categories provide improved capture of illness incidence where underreporting is likely, it comes at the cost of reduced reporting precision.

While Ecuador has experienced an epidemiologic transition in recent decades, the reduction of infectious causes of mortality and morbidity has been slow. Nationwide decreases in reported gastrointestinal infections have corresponded with the expansion of running water and wastewater services, but the rate of increase in sanitation and infrastructure access has been much slower in rural areas (PAHO, 2012). National surveys indicate that indigenous communities have the highest rate of unmet basic needs, including access to education and sanitation services (Ministerio Coordinador de Patrimonio UNICEF, 2010). Chimborazo has one of the highest proportions of indigenous residents in the country (INEC, 2010), and two-thirds (66.5%) of Chimborazo’s population lives below the poverty line (Instituto Nacional de Estadistica y Censos, 2014a), including areas in which >95% of the population is classified as having unmet basic needs (FAO, 2009). As with human disease estimates in Ecuador, few incidence estimates have been generated for either non-specific diarrhea or diagnosed gastrointestinal pathogens in livestock. Consequently, there is limited understanding of the potential association between human gastrointestinal infections and interaction with domestic animals on smallholdings (Zambrano et al., 2014). Transmission of many gastrointestinal pathogens might be effectively prevented in the context of livestock-keeping by modifying health behaviors and animal management practices. However, the influence that community norms and individual health beliefs may exert on reduction of zoonotic disease transmission is unknown. Understanding the perceptions of smallholder farmers regarding their health and that of their families and animals is critical in guiding planning efforts to reduce the incidence of pediatric diarrhea and improve the quality of life of those living in rural areas of Ecuador. Therefore, the objectives of this study were to: (a) assess self-reported personal and family health status and health concerns among smallholder farmers in Chimborazo province, Ecuador, (b) explore their animal and waste management practices, and (c) identify predictors of diarrheal illness among children and livestock in rural areas of Ecuador.

## Materials & Methods

### Study area and study population

This study was conducted in the Chimborazo Province of Ecuador, which is predominantly rural. Thirty-eight percent of the province’s population is comprised of indigenous residents making it the province with the highest population of this segment of the population (INEC, 2010). About a third (33.6%) of the residents are employed in agriculture (INEC, 2010), including crop cultivation and animal production. However, the agricultural capacity of communities in Chimborazo varies widely and is heavily influenced by elevation and annual rainfall. Dependence on livestock as a source of income is particularly common in areas >3,500 m above sea level, where few crops can be successfully grown (Córdova, Hogarth & Kanninen, 2018).

Chimborazo is divided into 10 counties (*cantones*) and further subdivided into parishes (*parroquias*). Located in the southeast corner of the province is the parish of Cebadas which is a pocket of extreme poverty, with an estimated 97.5% of residents living below the poverty line (Instituto Nacional de Estadistica y Censos, 2014a).

### Data collection and management

Sampling was conducted in three communities (San Antonio de Cebadas, Atillo, and Puca Totoras) of Cebadas Parish that have a total of 228 households. Based on provincial data indicating pediatric diarrhea prevalence of 16.1%, monthly pediatric prevalence of 2.0% was estimated (margin of error = .05). Cochran sample size calculations with a finite population correction were performed using Open Epi (Dean, Sullivan & Soe, 0000). A total of 27 respondents were required to achieve a 95% confidence level. Enrollment of additional respondents was desired in order to adequately represent households with children and permit inclusion of multiple predictors in regression analysis.

The three communities included in the study were located at or above 3,500 m above sea level and were accessible by vehicle most or all of the year. Residents of all three communities were predominantly Spanish-speaking or bilingual Spanish- and Quichua-speaking. Participant eligibility criteria included: household resident ≥18 years of age (preferably household head), able to consent to participation, and able to speak Spanish well or very well. Eligibility was assessed for each potential participant before the informed consent process was conducted. The survey was conducted in Spanish, with all participants enrolled and surveyed by a single interviewer in order to ensure consistency in survey conduct and response assignation. The informed consent document was read to participants and a copy provided to each for their records. Participants were encouraged to ask questions and were asked to verbally acknowledge their understanding of the study procedures before signing the informed consent document retained by the investigator.

Study participants were recruited during July/August 2019. Sampling of households was conducted in coordination with the president of each community. Consecutive houses were approached until an eligible individual was identified and agreed to participate. The survey was immediately conducted on-site. The adjacent neighbor of a participating household was skipped and then the process repeated, approaching consecutive households until identification and enrollment of another eligible individual. Enrollment was limited to a single adult resident in each household. Households were not re-approached if adult residents present at approach declined to participate.

Data were collected through structured interviews which gathered information on demographic characteristics of household members, land ownership and characteristics of properties owned, and the role of remittances as a portion of household income. A range of questions were included regarding the number and type of household-owned animals and details of animal interactions engaged in by either the respondents or pediatric household members. Respondents were asked about episodes of diarrheal illness experienced by household members and household-owned animals in the 30 days prior to interview. A diarrheal episode was defined as ≥3 watery stools within a 24-hour period. For participants reporting diarrhea, follow-up questions gathered details regarding how the illness was addressed or treated. Finally, information was elicited on health concerns as well as suggestions regarding potential interventions to address them. Data were collected electronically using Epi Info 7 (Centers for Disease Control and Prevention, Atlanta, GA), which was also used for exploratory review.

### Descriptive analysis

Analyses of the survey data were conducted in SAS 9.4 (SAS Institute, Cary, NC, USA). Variables collected were largely dichotomous or categorical in nature. Descriptive analyses included demographic characterization of households and assessment of associations of household characteristics with health concerns, pediatric health status, and health behaviors. The principal dependent/outcome variables assessed were reported pediatric and livestock diarrhea. Percentages of responses in each level of categorical variables and their 95% confidence intervals were also computed.

Chi-squared analyses were used to assess the relationship between the dependent variables (pediatric and livestock diarrhea) and categorical independent variables. Independent variables assessed included respondent factors (educational attainment), household factors (total household residents, running water in home, indoor toilet, household receipt of remittances), smallholding factors (property adjacency, size, running water availability, running water type, animal enclosures, animal waste management system), and pediatric factors (consumption of unpasteurized milk, consumption of untreated water, exposure to recreational water sources, participation in animal butchering, participation in milking dairy cattle, and interaction with animal waste). Pediatric diarrhea was also assessed for association with livestock diarrhea.

### Univariable and multivariable models

Potential predictors of pediatric and livestock diarrhea were first identified by fitting a series of univariable logistic regression models. Independent variables with univariable *p*-values ≤.20 were considered for assessment in the multivariable models. Subsequent to the univariable analyses, two multivariable logistic regression models (pediatric and livestock diarrhea) were fitted using a backwards stepwise approach with a critical *p*-value of .05. Since pediatric diarrhea was a relatively rare outcome, the Firth logistic model was used to investigate its predictors in order to correct for small-sample bias associated with maximum likelihood estimation of the ordinary logistic regression model. However, an ordinary logistic model was used for livestock diarrhea. Confounders were assessed by running the models with and without the suspected confounder and evaluating the changes in coefficients of the variables remaining in the models. Changes of 20% or greater indicated confounding and hence the variable was retained in the model, regardless of significance level. Odds ratios (ORs) and their 95% confidence intervals (95 CIs) were calculated for all variables included in the final models. Goodness-of-fit of the logistic regression models was assessed using the Hosmer-Lemeshow test.

### Ethics approval

Approvals for study implementation were granted by both the University of Tennessee Institutional Review Board (IRB Number 19-05199-XM) and the Pontifical Catholic University of Ecuador Committee for Ethics in Human Subjects Research (Approval Number 2019-95-EO).

## Results

### Demographic and household characteristics

A total of 58 respondents participated in the study. Self-identified race of the respondents was principally Indigenous (65.5%, 38/58) (Table 1). Many respondents (56.9%, 33/58) reported their primary language to be Spanish, but more than a quarter (27.6%, 16/58) spoke both Spanish and Quichua. Most (82.8%, 48/58) of the respondents owned their land, while the rest either farmed on property belonging to a family member (13.8%, 8/58) or rented the smallholding (1.7%, 1/58). A total of 70.7% (41/58) of the respondents reported having completed primary school education while 3.4% (2/58) reported having completed secondary school or higher level of education.

**Table 1:** Characteristics of respondents and households from Cebadas Parish, Chimborazo Province, Ecuador, 2019.

	**Percent** **(*n* = 58)**	**95% CI** ^a^
**Demographic Characteristics**		
**Race**		
Mestizo	32.8	22.1–45.6
Indigenous	65.5	52.7–76.4
**Primary language**		
Spanish	56.9	44.1–68.8
Quichua	15.5	8.4–26.9
Spanish/Quichua equally	27.6	17.8–40.2
**Land ownership**		
Owned land used for agriculture	82.8	71.1–90.4
Land used for agriculture belonged to a family member	13.8	7.2–24.9
Rented the land used for agriculture	1.7	0.3–9.1
**Educational attainment**		
Did not complete primary school	25.9	16.4–38.4
Completed primary, but did not complete secondary school	70.7	58.0–80.8
Completed secondary school or higher	3.4	1.0–11.7
**Household Characteristics**		
**Households with residents in age group**		
<5 years	22.4	13.7–34.7
5–12 years	62.1	49.2–73.4
13–17 years	51.7	39.2–64.1
Adults (≥18, <65 years)	98.3	90.9–99.7
Seniors (≥65 years)	19.0	10.9–30.9
**Households with >2 Adult contributors to household income**	17.2	9.6–28.9
**Households with non-resident contributors to household income**	3.4	1.0–11.7
**Households with ≥1 adult resident employed in agriculture**	94.8	85.9–98.2
**Principal smallholding location**		
Adjacent to house (median size = 2.0 hectares)	75.9	63.5–85.0
Non-adjacent parcel (median size = 2.5 hectares)	24.1	15.0–36.5

**Notes.**

a Confidence interval.

The median age of respondents was 39.5 years (interquartile range: 32, 50). Although total household size ranged from 1 to 11 members, the median household size was 4 (interquartile range: 3, 6). Most households comprised a bi-generational family unit including either middle-aged adults with children or seniors with unmarried adult children (Table 1). Multi-generational households were also noted, although extended families sharing resources and domestic responsibilities more commonly inhabited adjacent houses, rather than a single-family home. Sources of income outside the household were reported by 3.4% (2/58) of the respondents (Table 1). Both households reporting such remittances received less than 10% of total household income from the non-resident contributors.

A high proportion (80.3%, 122/152) of adult household residents were reported to be employed in agriculture, representing 94.8% of all households (Table 1). Regardless of employment type or status, all respondents reported either cultivating crops or raising animals on one or more plots of land. While these varied widely in size, from a micro-parcel of 200 square-meters (0.02 hectares) to a property of 20 hectares, a large proportion (75.9%, 44/58) of respondents reported that their principal smallholding was adjacent to their home (Table 1). The median size of adjacent smallholdings was 2.0 hectares (interquartile range: 0.64, 3.0). A smaller proportion of respondents had non-adjacent smallholdings, with a median size of 2.5 hectares (interquartile range: 1.0, 4.0). For those respondents with a primary smallholding site non-adjacent to their home, the median travel distance was 1 Km (interquartile range: 0.5, 2).

### Animal ownership and husbandry

The respondents reported a number of animal species reared on the smallholdings (Table 2). Nearly all respondents kept cattle (98.3%) and pigs (97.8%) (Table 2). Respondents’ principal revenue source was dairy or mixed dairy/beef production. Eighty-one percent of the respondents reported keeping cattle on the holding contiguous to the home while 17.3% kept cattle on holdings not contiguous to the land where the home was built. Only large animals (cattle, donkeys, and horses) were kept on non-adjacent smallholdings. Numbers of cattle owned varied widely among respondents, from single cow/calf pairs to multiple head of cattle in beef operations. The median number of cattle owned was 8 (interquartile range: 6, 10) (Table 2). Nearly three-quarters (72.4%, 42/58) of the respondents reported that their smallholdings did not have animal enclosures. All respondents who reported having enclosures used barbed-wire (93.8%, 15/16) or electric (6.2%, 1/16) fencing, while those without fencing reported tethering their livestock to pasture or allowing them to roam free. None of the respondents reported having a barn or similar structures for livestock.

**Table 2:** Household-owned animals in Cebadas Parish, Chimborazo Province, Ecuador, 2019.

**Animal type and location**	**Percent of households** (*n* = 58)	**95% CI** ^a^	**Median number**	**Quartile 1**	**Quartile 3**
**Household**					
Dogs	86.2	75.1–92.8	1	1	2
Cats	60.3	47.5–71.9	1	0	1
Chickens	87.9	77.1–94.0	4.5	2	6
Rabbits	41.4	29.6–54.2	0	0	3
Guinea Pigs	84.5	73.1–91.6	10	3	20
Pigs	97.8	90.9–99.7	1	1	2
Sheep	56.9	44.1–68.8	2	0	5
Cattle	81.0	69.2–89.1	6	3	10
Donkeys	34.5	23.6–47.3	0	0	1
Horses	44.8	32.8–57.6	0	0	2
**Smallholding**					
Cattle	37.9	26.6–50.8	0	0	4
Donkeys	3.5	0.01–11.7	0	0	0
Horses	3.5	0.01–11.7	0	0	0
**Total**					
Cattle	98.3	90.9–99.7	8	6	10
Donkeys	37.9	26.6–50.8	0	0	1
Horses	48.3	35.9–60.8	0	0	2

**Notes.**

a Confidence interval.

### Sanitation and waste management characteristics

Fewer than half (41.4%, 24/58) of the respondents reported having indoor running water. The largest proportion of respondents had only outdoor toilets (36.2%, 21/58), while 22.4% (13/58) reported having only indoor toilets and 6.9% (4/58) had both indoor toilets and pit latrines (Table 3). About a quarter (27.6%, 16/58) of the households had only pit latrines while 6.9% (4/58) had neither toilets nor latrines.

**Table 3:** Water and sanitation characteristics of households and smallholdings in Cebadas Parish, Chimborazo Province, Ecuador, 2019.

**Characteristic**	**Percent**	**95% CI** ^a^
**Household**		
**Indoor running water**	41.4 (24/58)	29.6–54.2
**Availability of toilets/latrine**		
Outdoor bathroom (toilet) only	36.2 (21/58)	25.1–49.1
Indoor bathroom (toilet) only	22.4 (13/58)	13.6–34.7
Both indoor toilet and outdoor latrine	6.9 (4/58)	2.7–16.4
Outdoor pit latrine only	27.6 (16/58)	17.8–40.2
No toilet or latrine	6.9 (4/58)	2.7–16.4
**Smallholding**		
**Irrigation system on-site**	48.2 (27/56)	35.7–61.0
**Running water**		
Piped	17.2 (10/58)	9.6–28.9
Surface water	55.2 (32/58)	42.5–67.3
No running water on-site	27.6 (16/58)	17.8–30.2
**Availability of toilet/latrine**		
Indoor bathroom (toilet)	3.6 (2/55)	1.0–12.3
Outdoor latrine	5.4 (3/55)	1.8–14.6
**Human waste management**		
No human waste management	10.3 (6/58)	4.8–20.8
Burn/bury onsite	29.3 (17/58)	19.2–42.0
Cover with grass	22.4 (13/58)	13.6–34.7
Leave exposed	17.2 (10/58)	9.6–28.9
Wait or return home	15.5 (9/58)	8.4–26.9
Use river/stream	1.7 (1/58)	0.3–9.1
Other response	3.5 (2/58)	1.0–11.7
**Animal waste management plan**		
Wash down/spread manure	12.1 (7/58)	6.0–22.9
Wastewater holding pond	3.5 (2/58)	1.0–11.7
No waste management plan	84.5 (49/58)	73.1–91.6
**Animal Waste Use**		
Fertilize pasture	63.8 (37/58)	50.9–75.0
Fertilize crops	10.3 (6/58)	4.8–20.8
No specified use	25.9 (15/58)	16.4–38.4

**Notes.**

a Confidence interval.

Most (75.9%, 44/58) of the smallholdings were unimproved, lacking waste management systems for both human and animal waste. Just under half (48.2%) of the smallholdings had on-site irrigation systems while 72.4% (42/58) had running water via either piped municipal water (17.2%) or surface water (55.2%) sources (Table 3). However, commensurate with the low proportion of piped water on smallholdings (17.2%, 10/58), very few respondents reported having a toilet (3.6%, 2/55) or latrine (5.4%, 3/55) onsite at the smallholding. In the absence of sanitary sewer systems, most smallholdings relied on improvised human waste management techniques, including burning/burying (29.3%, 17/58), covering with grass (22.4%, 13/58), and using rivers or streams for defecation (1.7%, 1/58) (Table 3). Similarly, respondents reported very limited use of animal waste management systems, with just two respondents (3.5%) reporting the use of a wastewater holding pond. Consistent with the proportion of respondents reporting no waste management plan (84.5%, 49/58), most smallholdings (74.1%, 43/58) relied on regular spreading of animal manure on pasture (63.8%, 37/58) or crops (10.3%, 6/58) to manage animal waste.

### Prevalence of diarrhea

In the preceding 30 days, 12.1% of the respondents had experienced diarrhea, yet very few (28.6%, 2/7) of these had sought care for their illness (Table 4). In 15.2% (7/46) of households in which children lived, one or more children had diarrhea in the previous month. Children with diarrhea ranged in age from 1 to 13 years of age with a median age of 5 years (interquartile range: 1, 11). *Post hoc* power of 0.936 was calculated for detection of the observed pediatric diarrhea prevalence. There was no significant (*p* = .48) difference in the median number of days of diarrhea experienced by children and adult respondents. A much higher proportion (71.4%, 5/7) of respondents sought care for the child or children that experienced diarrhea than for their own diarrhea. At least one animal in 55.2% of the households was reported to have experienced diarrhea in the previous 30 days. Despite the high frequency of diarrheal illness among livestock, fewer than half of these respondents (40.6%, 13/32) reported having sought veterinary care for their animals because of the diarrhea. Respondents indicated that, when sought, animal care was usually obtained from more skilled community members or para-veterinary professionals.

**Table 4:** Reported diarrheal events, care-seeking, and potential exposure sources among household members from Cebadas Parish, Chimborazo Province, Ecuador, 2019.

**Household member**	**Percent**	**95% CI** ^a^
**Adult Respondent**	*n* = 58	
**Diarrheal event in previous 30 days**	12.1	6.0–22.9
Sought medical care for diarrhea	28.6 (2/7)	8.2–64.1
**Food- and Water-borne exposures**	91.4	81.4–96.3
Drinks unpasteurized milk	22.4	13.6–34.7
Drinks untreated water	74.1	61.6–83.7
Bathes/uses untreated water	53.4	40.8–65.7
**Fecal- or Blood-borne exposures**	100.0	93.8–100.0
Participates in butchering animals	60.3	47.5–71.9
Milking dairy cattle	98.3	90.9–99.7
Handles animal waste	87.9	77.1–94.0
**Any Children in Household**	*n* = 46	
**Diarrheal event in previous 30 days**	15.2	7.6–28.2
<5 years of age	42.9 (3/7)	–
Sought medical care for diarrhea	71.4 (5/7)	35.9”—91.8
**Food- and Water-borne exposures**	93.5	82.5–97.8
Drink unpasteurized milk	21.7	12.3–35.6
Drink untreated water	80.4	66.8–89.4
Bathe/use untreated water	56.5	42.3–69.8
**Fecal- or Blood-borne exposures**	80.4	66.8–89.4
Participate in butchering animals	41.3	28.3–55.7
Milking dairy cattle	76.1	62.1–86.1
Handle animal waste	63.0	48.6–75.5
**Any Household-Owned Animal**	*n* = 58	
**Diarrheal event in previous 30 days**	55.2	42.5–67.3
Sought medical care for diarrhea	40.6 (13/32)	25.5–57.7

**Notes.**

a Confidence interval.

### Potential sources of pathogen exposure

Exposures to potential sources of gastrointestinal pathogens were widespread. The percentage (93.5%) of children that was exposed to food- and water-borne potential sources of pathogens (drinking unpasteurized milk, drinking untreated water, and bathing or playing in surface water) was not significantly (*p* = .61) different from that of adults (91.4%). However, the percentage of children (80.4%) exposed to blood-borne and fecal transmission exposures (butchering animals, milking dairy cattle, and handling animal waste) was significantly (*p* < .0001) lower than that of adults (100.0%) (Table 4). The most common exposure categories among adults were (in rank order): milking dairy cattle (98.3%, 57/58), handling animal waste (87.9%, 51/58), and drinking untreated water (74.2%, 43/58). The categories of exposure most frequently reported for children were the same, but varied in rank: drinking untreated water (80.4%, 37/46), milking dairy cattle (76.1%, 35/46), and handling animal waste (63.0%, 29/46) (Table 4).

### Reported health concerns

Respondents reported specific concerns regarding their own health more frequently than any other personal health concern, with 55.2% (32/58) naming a particular disease or condition which they had experienced or about which they worried (Table 5). Specific concerns regarding family health were reported by 58.6% (34/58) of the respondents, but relatively few (29.4%, 10/34) of the family concerns specified pediatric health issues. One-fifth of respondents expressed concern regarding healthcare access for themselves (20.7%, 95% CI [12.3–32.8]) but only half as many reported such concern for family members (10.3%, 95% CI [4.8–20.8]). However, among households with cases of pediatric diarrhea, the proportion of respondents expressing concern about access to healthcare rose to 42.9% (3/7, 95% CI [15.8–75.0]). Concern regarding stress or mental health of either self or family members was reported by just 10.3% (6/58) of the respondents. In comparison, concern for physiological stress of animals was reported by 60.3% (35/58, 95% CI [47.5–71.9]) of respondents, ranking as the most frequently cited animal health concern (Table 5). Respondents listed a range of potential causes for illness in their livestock, including both general categories of disease and specific pathogens, as well as climatic and environmental influences (*e.g.*, “altitude sickness”). The respondents reported high levels of concern regarding the impact of health concerns, particularly stress, on their animals’ productivity. However, households reporting animal diarrhea did not demonstrate a greater level of concern regarding healthcare access than all households (Table 5).

**Table 5:** Reported health concerns for self, family, and animals, in Cebadas Parish, Chimborazo Parish, Ecuador, 2019.

**Domain**	**Category of concern**	**Respondents expressing concern** **(*n* = 58)** **–percent** **(95% CI** ^a^ **)**	**Households with pediatric diarrhea event expressing concern (*n* = 7)** **–percent** **(95% CI** ^a^ **)**	**Households with animal diarrhea event expressing concern (*n* = 32)** **–percent** **(95% CI** ^a^ **)**
**Self**	Access/Availability of health care	20.7 (12.3–32.8)	42.9 (15.8–75.0)	28.1 (15.6–45.4)
	Specified Illness	55.2 (42.5–67.3)	28.6 (8.2–64.1)	59.4 (42.3–74.5)
	Unspecified Illness	24.1 (15.0–36.5)	14.3 (2.6–51.3)	15.6 (6.9–31.8)
	Stress/Mental Health	10.3 (4.8–20.8)	0 (–)	12.5 (5.0–28.1)
	General Wellbeing/None	32.8 (22.1–45.6)	42.9 (15.8–75.0)	28.1 (15.6–45.4)
**Family**	Access/Availability of health care	10.3 (4.8–20.8)	42.9 (15.8–75.0)	15.6 (6.9–31.8)
	Specified Illness	58.6 (45.8–70.4)	57.1 (25.1–84.2)	62.5 (45.3–77.1)
	Unspecified Illness	25.9 (16.4–38.4)	14.3 (2.6–51.3)	32.3 (18.0–48.6)
	Stress/Mental Health	10.3 (4.8–20.8)	0 (–)	12.5 (5.0–28.1)
	General Wellbeing/None	27.6 (17.8–40.2)	28.6 (8.2–64.1)	21.9 (11.0–38.8)
**Animal**	Access/Availability of health care	22.4 (13.6–34.7)	57.1 (25.1–84.2)	25.0 (13.3–42.1)
	Specified Illness	39.7 (28.1–52.5)	28.6 (8.2–64.1)	40.6 (25.5–57.7)
	Unspecified Illness	44.8 (32.8–57.6)	14.3 (2.6–51.3)	37.5 (22.9–54.8)
	Stress	60.3 (47.5–71.9)	42.9 (15.8–75.0)	62.5 (45.3–77.1)
	General Wellbeing/None	15.5 (8.4–26.9)	28.6 (8.2–64.1)	15.6 (6.9–31.8)

**Notes.**

a Confidence interval.

### Predictors of pediatric and animal diarrhea

Results of both the univariable and final models investigating the associations between potential predictors of pediatric and animal diarrhea are shown in Table 6. Variables that had potential univariable associations (critical *p* ≤ .20) with pediatric diarrhea were: household with indoor running water (OR = 4.0; 95% CI [0.7–23.3]; *p* = .12), diarrhea in any household-owned animal (OR = 5.7; CI [0.6–51.9]; *p* = .12), any child in household consumes raw milk (OR = 3.4; CI [0.6–18.9]; *p* = .16), and cattle density per hectare at the smallholding (OR = 1.9; CI [0.9–4.0]; *p* = .11). None of the variables had a significant association with pediatric diarrhea at a critical *p* ≤ .05 and, therefore, none were retained in the final model.

**Table 6:** Univariable and Final Models for Pediatric and Animal Diarrhea in Cebadas Parish, Chimborazo Province, Ecuador, 2019.

**Predictor variable**	**Univariable models** **Odds ratios**	**95% CI** ^a^	*p*-value^*^	**Final models** **Odds ratios**	*p*-value^**^
**Pediatric model**				*No variables retained*	
Respondent education less than primary (vs. completed primary)	0.38	0.04–3.47	.39	–	–
Indoor running water (vs. No)	4.00	0.69–23.30	**.12**	–	–
Indoor bathroom (vs. No)	0.90	0.15–5.31	.91	–	–
Diarrhea in household-owned animal (vs. No)	5.70	0.63–51.86	**.12**	–	–
Any child in household consumes raw milk (vs. No)	3.43	0.62–18.88	**.16**	–	–
Any child in household consumes untreated water (vs. No)	4.67	0.21–104.17	.33	–	–
Any child in household bathes/plays in surface water (vs. No)	2.14	0.37–12.41	.40	–	–
Any child in household milks dairy cattle (vs. No)	0.75	0.12–4.54	.75	–	–
Any child in household handles animal waste (vs. No)	0.75	0.15–3.83	.73	–	–
Cattle density per hectare at household	1.06	0.93–1.22	.39	–	–
Cattle density per hectare at smallholding	1.86	0.87–3.98	**.11**	–	–
**Animal model**				*One variable retained*	
Smallholding running water (vs. No)	1.06	0.33–3.46	.92	–	–
Animal enclosure on smallholding (vs. No)	3.3	0.91–11.91	**.07**	–	–
Animal waste management plan (vs. No)	8.70	1.01–75.00	**.05**	**8.70**	**.049**
Cattle density per hectare at household	1.06	0.95–1.17	.31	–	–
Cattle density per hectare at smallholding	1.21	0.86–1.71	.28	–	–

**Notes.**

a Confidence interval.

* Univariable associations with *p*-value <.20 used as cutoff for assessment in multivariable models. Variables meeting this criterion are in bold.

** Associations with *p*-value <.05 were retained in the final models. Variables meeting this criterion are in bold.

Variables that had potential univariable associations (critical p ≤.20) with animal diarrhea were: animal enclosure on smallholding (OR = 3.3; CI [0.9–11.9]; *p* = .07) and animal waste management plan for smallholding (OR = 8.7; CI [1.0–75.0]; *p* = .049). The latter was the only significant predictor in the final animal diarrhea model (Table 6).

## Discussion

This study assessed the health status and health concerns of smallholder farmers, explored their animal and waste management practices, and investigated predictors of diarrheal illness among children and livestock in rural areas of Ecuador. The near universal (98.3%) ownership of cattle by respondents in the present study is an indication of the importance of livestock to smallholder farmers in the region. Ecuador is a primarily agrarian nation in which traditional cultivation and animal production methods continue to be widely employed. While a study of rural Ecuadorians by the Food and Agriculture Organization of the United Nations (2015) (FAO) reported that 84% of respondents owned livestock (Zezza et al., 2007), the higher proportion found in the current study corresponds to that described by Kristjanson et al. in a study of Peruvian smallholders at similarly high altitudes (Kristjanson et al., 2007). Indeed, Kristjanson identified a marked difference in the importance of livestock to farmers’ livelihoods at different elevations, even while the size of landholding remained constant (Kristjanson et al., 2007).

All respondents in the present study kept their livestock on pasture, yet relatively few (27.6%) reported keeping them within enclosures of any type. Dairy cattle kept under such grazing systems require ample pasture to maintain production (Hodgson, 1990). However, the households in the current study, with a median of 8 head of cattle, relied on relatively small parcels of land. This increases the pressure on pastureland, risk of overgrazing (Pulido et al., 2018), and risk of infestation/re-infestation with gastrointestinal parasites. To avoid pasture depletion, respondents reported using traditional management practices such as transhumance (for non-dairy herds) and tether-grazing. Aubron (2006) have identified tether-grazing as one of the principal systems used in the Andes. Unfortunately, the utility of tethering is limited due to the time required to re-position animals multiple times a day if pastures are located at a distance from the household (Aubron et al., 2009). Thus, the practice appears more feasible for smallholders focused solely on dairy cattle production than those with a more diversified farming practice. Although tethering of cattle is practiced in many parts of the world, research on the practice’s role in the livestock-human disease cycle is scarce. Studies have focused on disease transmission associated with tethered pigs, particularly in areas with low rates of latrine availability (Flisser et al., 2003; Millogo, Kongnyu Njamnshi & Kabwa-PierreLuabeya, 2018). Nonetheless, studies have shown that potential for zoonotic disease exposure from a range of livestock is associated with management strategies and, therefore, the impact of livestock management practices on disease transmission warrants more investigation (Zambrano et al., 2014; Thumbi et al., 2015; Conan et al., 2017).

The availability of improved water sources (*i.e.*, piped potable water) varied widely across households and smallholdings. The proportion of households with running water (41.4%) in this study was similar to that reported by Odoi et al. among Kenyan smallholders (45.5%) raising small ruminants (Odoi et al., 2008). However, the reported proportion differs significantly from that of households with public utility-provided running water in Chimborazo overall, reported by INEC to be 62.9% as of 2010 (Instituto Nacional de Estadistica y Censos, 2010). Handwashing after using the toilet/latrine was unanimously reported in the present work, even in the absence of improved water sources, suggesting some understanding of disease exposure risk mitigation. The low percentage of smallholdings in the current study with toilets/latrines and improved water sources may explain the lower percentage (68.4%) of respondents reporting handwashing after using the toilet/latrine when at their smallholding compared to when they are at home.

Beyond incomplete household water access, availability of effluent handling systems was also deficient. Use of outdoor pit latrines and open defecation were more common in the current study than expected, while the availability of indoor toilets was much lower (36.2%) than the overall provincial proportion (77.7%) (Instituto Nacional de Estadistica y Censos, 2010). High reported availability of indoor toilets obscures unequal access to sanitary management systems. For instance, whereas the proportion of the population with access to public sewage systems is 47.4% across all of Chimborazo, (Instituto Nacional de Estadistica y Censos, 2010) only 15% of rural provincial households are connected to these (PAHO, 2012). Nationally, the proportion of homes in Ecuador with indoor toilets steadily increased over a 20-year period, reaching 89.3% by 2010 (Instituto Nacional de Estadistica y Censos, 2010). The increase in household toilets coincided with a decrease in diarrheal illness over the same period (Instituto Nacional de Estadistica y Censos, 2015a), indicating potential association between toilet/latrine availability and pathogen transmission. However, no association was noted in the present study between the availability of indoor toilets and pediatric diarrhea.

The reported 30-day prevalence of diarrhea among children (15.2%) was comparable to that previously reported in both Chimborazo (16.0%) (Instituto Nacional de Estadistica y Censos et al., 2015) and Ecuador (17.0%) (Instituto Nacional de Estadistica y Censos, 2015a). It should be noted that the prevalence estimated in the present study likely underrepresents the true burden among children since male household heads may be less involved in childcare and may be unaware of diarrheal episodes. A systematic review by Fischer Walker et al. of pediatric diarrhea studies in 139 low- and middle-income countries (LMICs) reported an incidence rate of 2.9 diarrheal episodes per child-year among children under five years of age (Fischer Walker et al., 2012). This represents a 14.7% reduction in incidence between 1990 and 2010, whereas Ecuador reported a 64.8% decrease in period prevalence during a comparable timeframe (Instituto Nacional de Estadistica y Censos, 2015a). Concurrent with this reduction in risk, the severity of diarrheal diseases among children under age 5 also appears to be decreasing in Ecuador (Institute for Health Metrics and Evaluation, 2010; Moncayo et al., 2019). Moncayo et al. (2019) reported a 1.9% reduction in diarrheal hospitalizations and a 78.6% decrease in diarrheal deaths in this age group between 2009 and 2014, which correlated with improvements in socioeconomic factors at a county level. The 30-day household prevalence of diarrhea among livestock (55.2%) in the present study is more difficult to compare. Thumbi et al. (2015) identified gastrointestinal illnesses, principally characterized by diarrheal episodes, as responsible for 56% of all syndromic illness reports among participants’ livestock.

The high percentages of children (93.5%) and adults (91.4%) exposed to at least one potential source of food- and water-borne pathogens (drinking unpasteurized milk, drinking untreated water, and bathing or playing in surface water) demonstrates the structural limitations of the communities. In contrast, the significantly different percentages of children (80.4%) and adults (100.0%) exposed to at least one blood-borne and fecal transmission exposure type (butchering animals, milking dairy cattle, and handling animal waste) reflects the differences in household responsibilities. Although wide variability was noted between different exposures, the median number of exposure types (4) for both children and adults indicates multiple potential routes of zoonotic pathogen infections exist. Exposure to multiple sources is consistent with the findings of other studies of smallholders (Herrero et al., 2013; Helmy et al., 2014; Tebug et al., 2015; Kelly et al., 2018), however, the specific sets of exposures likely vary with the context. A study of Senegalese cattle farmers by Tebug et al. found that 95% consumed unpasteurized milk and 98% of those assisting in birth procedures did not wash their hands afterward (Tebug et al., 2015). In contrast, in Nepal, smallholder farmers studied by Kelly et al. reported near unanimous consumption of pasteurized milk and handwashing after interactions with animals, but fewer than 40% reported regular treatment of drinking water (Kelly et al., 2018).

While most respondents used cattle manure for fertilizing pastures, sheep and guinea pig waste were applied to crops intended for human consumption. Work by Kelly et al. with Nepali farmers reported 13% applied manure directly to their fields (Kelly et al., 2018), although the study did not specify if these were pasture, crop, or mixed used applications, nor if the farmers selectively applied manure by type. Manure spreading has been associated in previous studies with increased respiratory (Phillips et al., 2003) and gastrointestinal (Moussavou-Boussougou et al., 2005) illness in cattle. Washing the manure down in a pasture setting, which was the most frequently reported method of manure spreading in the present study, may have the same effect as that of runoff following heavy rainfall (Soupir et al., 2006), which increases the possibility of pathogen contamination of surrounding pasture land.

The significantly higher odds of animal diarrhea (OR = 8.7; 95% CI [1.0–75.0]; *p* = .049) among households that had animal waste management plans compared to those that did not have such plans may reflect the level or frequency of the herds’ exposure to manure. Whereas households that reported having a manure management strategy were those that also had animal enclosures and typically washed down or manually spread the manure, households without a manure management plan relied on tethered grazing. Rotational tethering (tying animals in a different grazing location each day) may have inadvertently reduced the risk of manure (and hence parasite) exposure for tethered cattle compared to those that were enclosed. Association of tethering with helminthiasis has been previously reported by Nalubwama et al. in a study of mixed production smallholder farms in Uganda (Nalubwama et al., 2016).

The respondents in this study reported a wide range of health concerns and although many conditions they reported could not be treated in the communities, few of the smallholders listed access/availability of healthcare among their top concerns. A similarly wide range of perceived health risks were reported among Kenyan farmers by Anthonj et al. (2019). As in the present study, the Kenyan farmers named vector-borne disease risks, zoonotic diseases, environmental contaminants, infrastructure insufficiency, and a broad set of climatological pressures among their health concerns, but a varying proportion of smallholders expressed concerns tying the lack of healthcare to these exposures (Anthonj et al., 2019). In a recent systematic review of literature concerning the mental health of farmers, (Yazd, Wheeler & Zuo (2019) identified physical health or injury as a risk factor, although this was assessed in just five studies conducted in LMICs.

In the present study, households that reported children with diarrhea in the previous month were significantly more likely to identify availability of health services as a concern than households in which children had not had diarrhea (42.9% vs. 11.8%). In contrast, a study of caregivers by Luque et al. in a nearby area of rural Ecuador reported that care-seeking for pediatric illnesses was principally hindered by the cost of medicine or transport (Luque, Whiteford & Tobin, 2008), with smaller proportions of respondents reporting availability (clinic hours or distance) as a barrier. Since the study by Luque et al. was conducted in a healthcare setting, it is important to note that the barriers reported by caregivers seeking medical attention may differ from the barriers anticipated by respondents.

The large proportion (60.3%) of respondents reporting concern for physiological stress in their animals reflects the central importance of livestock to the smallholders’ livelihoods. Although internal parasites and climatic stresses were reported to affect both humans and animals, none of the respondents specifically mentioned zoonotic disease transmission, suggesting limited knowledge of the behaviors which might protect the health of the smallholder farmers and their families. A study of Nepali buffalo farmers by Kelly et al. found that a large proportion (45%) of participants did not know that diseases could be transmitted between animals and humans (Kelly et al., 2018). Likewise, Tebug et al. describe a lack of awareness regarding zoonoses among 68.9% of farmers surveyed in Senegal (Tebug et al., 2015), but among just 23% of dairy farmers in Malawi (Tebug et al., 2014). Furthermore, very few of the Senegalese farmers (11.3%) surveyed perceived their exposure to such pathogens to be likely (Tebug et al., 2015). In addition to identifying this type of knowledge gap, understanding smallholder health concerns is critical for the development of effective community-guided interventions.

### Strengths and limitations

Studies of health concerns of rural smallholding families in Latin American countries and other LMICs are scarce and therefore the present work provides a unique insight into the issues in these communities. The structured interview format elicited numerous topics of health concern that can be addressed in future community interventions. Additionally, this study considers both demographic factors and animal management practices associated with diarrhea prevalence, which have been poorly described in the literature to date. Use of the Firth logistic model is an analytical strength of the study as it corrects for small sample bias associated with ordinary logistic models. However, the study is not without drawbacks. The findings are based on respondent reports and therefore subject to recall biases, particularly related to reports of pediatric diarrhea and medical care sought. This could have been addressed by asking additional questions of the main caregivers of children. However, to minimize respondent burden (which would in turn impact response rate), the interview time had to be limited and questions prioritized. Study findings should be interpreted with caution, given the relatively low number of respondents involved in the study. Future studies should be conducted across a broader geographic area, implementing a multistage randomized sampling scheme to improve representativeness and generalizability. Confirmation of diarrheal illness through medical record evaluation or specimen collection should also be sought. These limitations notwithstanding, the findings of the current study contribute to the otherwise sparse body of literature on the health and livelihoods of rural smallholder Ecuadorian farmers and provide useful information to guide future larger studies of these populations in Ecuador and other LMICs.

### Conclusions

The levels of diarrheal illness among the children of smallholder families in this study is similar to national and international estimates. Ongoing surveillance is necessary to permit accurate identification of diarrheal illnesses among both household members and livestock and develop more detailed incidence estimates. A natural next step is to evaluate specific gastrointestinal pathogen prevalence and assess the role of livestock in specific pathogen disease burden among smallholder farmers and their children. This would help guide the development of economically feasible interventions that are technologically appropriate for the region. Mitigation of waste exposure through improvement of sanitation and waste management infrastructure is recommended to reduce the burden of diarrhea both in humans and livestock. Future studies must also involve community members in the design and implementation of educational interventions aimed at decreasing human and animal diarrheal diseases.

##  Supplemental Information

10.7717/peerj.12208/supp-1 Supplemental Information 1 English Translation of Survey Questionnaire

10.7717/peerj.12208/supp-2 Supplemental Information 2 Spanish Translation of Survey Questionnaire

10.7717/peerj.12208/supp-3 Supplemental Information 3 Research Data

10.7717/peerj.12208/supp-4 Supplemental Information 4 Data Dictionary

## Additional Information and Declarations

### Data Availability

The following information was supplied regarding data availability: The raw data with all variables collected from the study are available in the Supplementary Files.
