# Supplementary material for: An exploratory assessment of human and animal health concerns of smallholder farmers in rural communities of Chimborazo, Ecuador
Source: PeerJ. 2022 Jan 17;9:e12208. doi: 10.7717/peerj.12208 (PMC8772447; doi:10.7717/peerj.12208)
Supplement: Supplemental Information 1 [file peerj-10-12208-s001.pdf]

Survey: ***Assessment of human and animal health concerns in rural communities of Chimborazo, Ecuador***

Demographics

Thank you for agreeing to participate in this study. I'd like to start by getting to know a little bit about you and your family.

(HeadofHH) Are you the head of household? Y/N/DK/R

(AgeRpt) What is your age? ________

(Race-Eth) What race or races do you consider yourself? (check all that apply)
- Mestizo
- Indigenous/native
- Hispanic/Latino
- White
- Black/Afro-Ecuadoran
- Other: Specify ____________________________
- Don't know
- Refused

(PmryLang) What language do you speak most at home?
- Spanish
- Quichua
- Both Spanish and Quichua equally
- Other: Specify ____________________________

(Landowner) Do you own the land that you use for farming? Y/N/DK/R

Household makeup

(TotalHH) How many people live in your household, either full or part-time? ________
[Check box for Don't know/Released]

I would like to know more about the people living in your household. For each age category I mention, please tell me how many people in your household are in that age category. Also please tell me what type of job each person does.

Age Category	Number	Individual Occupations	Categories
Adults (>18 years, <65 years)<br>(HHcatAdult)		(Adult1Occ): ___________<br>(Adult2Occ): ___________<br>(Adult3Occ): ___________<br>(Adult4Occ): ___________<br>(Adult5Occ): ___________	Agriculture<br>Homemaker<br>Self-Employed<br>Professional<br>Disabled<br>Unemployed<br>Retired<br>Other
Seniors (≥65 years)<br>(HHcatSenior)		(Senior1Occ): __________<br>(Senior2Occ): __________<br>(Senior3Occ): __________	Agriculture<br>Homemaker<br>Self-Employed<br>Professional<br>Disabled<br>Unemployed<br>Retired<br>Other

<5 years<br>(HHcatLT5)		(LTF1Occc): ___________<br>(LTF2Occ): ___________<br>(LTF3Occ): ___________<br>(LTF4Occ): ___________	Student<br>Not in school yet
5-12 years<br>(HHcat512)		(Tween1Occ): _________<br>(Tween2Occ): _________<br>(Tween3Occ): _________<br>(Tween4Occ): _________	Student<br>Not in school<br>Working<br>Student+Working
13-17 years<br>(HHcat1317)		(Adol1Occ): _________<br>(Adol2Occ): _________<br>(Adol3Occ): _________<br>(Adol4Occ): _________	Student<br>Not in school<br>Working<br>Student+Working

Socioeconomic status proxies

(RespEduc) What is the highest level of school you have completed?

- No formal education
- Some primary school
- Completed primary school
- Some secondary school
- Completed secondary school
- Some university
- Completed university
- Post-graduate study
- Trade/vocational program
- Other: Specify____
- Refused

(Homeowner) Do you own or rent the property where you live?

- Own
- Rent
- Other: Specify _____________________________
- Don’t know/Refused

House details

(HHAldea) What is the name of the area where your house is located? (aldea/recinto/pueblo)

____

[Check box for Don’t know/Refused]

(HHBdrms) How many bedrooms are there in the house you live in? _________

[Check box for Don’t know/Refused]

(HHRunWtr) Does the house you live in have indoor running water? Y/N/DK/R

(HHInBath) Does the house you live in have an indoor bathroom? Y/N/DK/R

(HHInBathNum) If yes, How many indoor bathrooms does it have? _________

[Check box for Refused]

(HHLatrine) Does the house you live in have an outdoor latrine that is used? Y/N/DK/R

(HHWash) When you are at home, do you wash your hands after using the restroom/latrine? Y/N/DK/R

(HHWashLoc) If yes, Where do you wash your hands after using the latrine? ______

Smallholding details

(SHLoc) Is the house you live in part of the property that you farm? Y/N/DK/R

(SHAldea) What is the name of the area where your farm is located? (aldea/recinto)
____
[Check box for Don’t know/Refused]

(SHSize) What is the size of the property that you farm? ______________
[Check box for Don’t know/Refused]
(Calculate hectares from answer if given in other measure)

(SHDist) How far away is the property from the house that you live in? __________
[Check box for Don’t know/Refused]

(TimetoSH) How long does it take you to get there? (minutes) ________
[Check box for Don’t know/Refused]

(SHTrvlType) How do you typically travel to the farm?

- Walking
- Horse/donkey
- Car/truck
- Motorcycle/ATV
- Other: Specify__________________________________
- Don’t know/Refused

Now I’d like to ask you about the animals that you raise.

What type of animals do you have in your home or at the property your house is on?

**Type**	**Number**
Dog (HHDog)	
Cat (HHCat)	
Chicken (HHChkn)	
Duck/Goose (HHDuck)	
Other Poultry (HHPltry)	
Guinea Pig (HHCuy)	
Rabbit (HHRabbit)	
Sheep (HHSheep)	
Goat (HHGoat)	
Cattle (HHCattle)	
Donkey (HHDonkey)	
Horse (HHHorse)	
Llama (HHLlama)	
Alpaca/Other Camelid (HHAlpaca)	
Other (HHOthAnimal)	
Specify (HHOthAnSp): _________	

What type of animals do you have at your farm?

**Type**	**Number**
Dog (SHDog)	
Cat (SHCat)	
Chicken (SHChkn)	
Duck/Goose (SHDuck)	
Other Poultry (SHPltry)	
Guinea Pig (SHCuy)	

Rabbit (SHRabbit)	
Sheep (SHSheep)	
Goat (SHGoat)	
Cattle (SHCattle)	
Donkey (SHDonkey)	
Horse (SHHorse)	
Llama (SHLlama)	
Alpaca/Other Camelid (SHAlpaca)	
Other (SHOthAnimal)	
Specify (SHOthAnSp): __________	

(SHIrrig) Does the farm have water for irrigation? Y/N

(SHIrrigHow) If yes, How is it collected/distributed?

- Canal/Ditch
- Holding pond/Cistern/Rainwater collection barrels
- Municipal water system
- Natural spring (ojo de agua)
- Other: Specify____________________________________
- Don't know/Refused

(SHRunWtr) Does the farm have running water? Y/N/DK/R

(SHWtrTyp) If yes, Is it piped (municipal) or natural? Piped/Natural/DK/R

(SHInBath) Does the farm have an indoor restroom? Y/N/DK/R

(SHLatrine) Does the farm have a latrine? Y/N/DK/R

(SHHWash) When you are on the farm, do you wash your hands after using the restroom/latrine? Y/N/DK/R

(SHHWashLoc) If yes, Where do you wash your hands after using the latrine? ______

(SHHmWst) What do you do with the human waste on the farm?

- Use river/stream
- Bury it
- Burn it
- Holding pond
- Compost pile
- Biodigester
- Spread directly on crops
- Other: Specify_____________________________________
- Don't know/Refused

(SHHmWUse) [If yes to spread on crops] What type of crops do you use the waste on?

____

(SHAnEncl) [If yes to animals on farm] Does the farm have any animal enclosures? Y/N/DK/R

(SHEnclDes) If yes, describe: ____

(SHAnWst) [If yes to animals on farm] Does the farm have an animal waste handling system of any type? Y/N/DK/R

(SHWstHow) If yes, What type?

- Holding pond
- Compost pile
- Biodigester

Other: Specify___________________________________
Don't know/Refused

(AnWstUse) [If yes to animals on farm] What do you do use the waste for ultimately?
Spread directly on crops
Compost
Use in adobe
Burn it
No specific use/holding
Other: Specify__________________________
Don't know/Refused

Health concerns and behaviors

Now I'd like to ask you about some thoughts you may have about health.

(PerHlth) How would you classify your health?
Excellent
Good
Average
Poor
Very Poor
Refused

What are the three things that most concern you about your health?
(PerCrn1) ____
(PerCrn2) ____
(PerCrn3) ____

(StPerCrn) How would you classify these health concerns? Are these… (select all that apply)
Health issues you have currently?
Health issues you have had in the past?
Health issues someone in your family has currently?
Health issues someone in your family has had in the past?
Health issues someone else you know has currently?
Health issues someone else you know has had in the past?
Health issues that are common in the community?
Health issues you have heard about?
(via friends, acquaintances, media campaigns, medical providers, other)

What are the three things that most concern you about the health of your family?
(FamCrn1) ____
(FamCrn2) ____
(FamCrn3) ____

(StFamCrn) How would you classify these health concerns? Are these… (select all that apply)
Health issues you have currently?
Health issues you have had in the past?
Health issues someone in your family has currently?
Health issues someone in your family has had in the past?
Health issues someone else you know has currently?
Health issues someone else you know has had in the past?
Health issues that are common in the community?
Health issues you have heard about?
(via friends, acquaintances, media campaigns, medical providers, other)

What are the three things that most concern you about the health of your animals?

(AnmlCrn1) ____

(AnmlCrn2) ____

(AnmlCrn3) ____

(StAnCrn) How would you classify these health concerns? Are these… (select all that apply)

Health issues your animals have currently?
Health issues your animals have had in the past?
Health issues someone else's animals have currently?
Health issues someone else's animals have had in the past?
Health issues that are common in the community?
Health issues you have heard about?
(via friends, acquaintances, media campaigns, veterinarians, other)

I'd like to ask you a few questions about some ways that you and the children in your household interact with your animals.

(RawMAdlt) Do you drink unpasteurized milk? Y/N/DK/R

(RawMPed) Do any children in your household drink unpasteurized milk? Y/N/DK/R

(RawWAdlt) Do you drink untreated/unboiled water? Y/N/DK/R

(RawWPed) Do any children in your household drink untreated/unboiled water? Y/N/DK/R

(RecWAdlt) Do you bathe, play, or wash in water from lakes, rivers, creeks, canals, or other natural sources? Y/N/DK/R

(RecWPed) Do any children in your household bathe, play, or wash in water from lakes, rivers, creeks, canals, or other natural sources? Y/N/DK/R

(BtchAdlt) Do you participate in butchering animals? Y/N/DK/R

(BtchPed) Do any children in your household participate in butchering animals? Y/N/DK/R

(MilkAdlt) Do you milk animals? Y/N/DK/R

(MilkPed) Do any children in your household milk animals? Y/N/DK/R

(AnWstAdlt) Do you interact with animal waste? (pen cleaning, composting, etc.) Y/N/DK/R

(AnWstPed) Do any children in your household interact with animal waste? (pen cleaning, composting, etc.) Y/N/DK/R

(AdltDiar) In the past month, have you had any episodes of diarrhea? Y/N/DK/R
(AdltDDays) If yes, How many days did the diarrhea last? __________
(AdltDCare) If yes, Did you seek medical care? Y/N/DK/R
(AdltDDx) If yes, Did you receive a diagnosis for the diarrhea? Y/N/DK/R
(AdltDCause) If yes, What was the diagnosis? _______________
[Check box for Don't know/Refused]

(PedDiar) In the past month, have any children in the household had any episodes of diarrhea? Y/N/DK/R
(PedDEpis) If yes, How many of the children? __________
[If >3 had diarrhea, capture the three youngest children's details]

(Ped1DAge) If yes, What is the age of the child? __________
(Ped1DDay) If yes, How many days did the diarrhea last? __________
(Ped1DCare) If yes, Did you seek medical care for them? Y/N/DK/R
(Ped1DDx) If yes, Did you receive a diagnosis for their diarrhea? Y/N/DK/R
(Ped1DCse) If yes, What was the diagnosis? ____________
[Check box for Don't know/Refused]

(Ped2DAge) If yes, What is the age of the child? __________
(Ped2DDay) If yes, How many days did the diarrhea last? __________
(Ped2DCare) If yes, Did you seek medical care for them? Y/N/DK/R
(Ped2DDx) If yes, Did you receive a diagnosis for their diarrhea? Y/N/DK/R
(Ped2DCse) If yes, What was the diagnosis? ____________
[Check box for Don't know/Refused]

(Ped3DAge) If yes, What is the age of the child? __________
(Ped3DDay) If yes, How many days did the diarrhea last? __________
(Ped3DCare) If yes, Did you seek medical care for them? Y/N/DK/R
(Ped3DDx) If yes, Did you receive a diagnosis for their diarrhea? Y/N/DK/R
(Ped3DCse) If yes, What was the diagnosis? ____________
[Check box for Don't know/Refused]

(AnDiar) In the past month, have any animals in the household or on your farm had any episodes of diarrhea? Y/N/DK/R
(AnDDays) If yes, How many days did the diarrhea last? __________
(AnDCare) If yes, Did you seek veterinary care for them? Y/N/DK/R
(AnDDx) If yes, Did you receive a diagnosis for their diarrhea? Y/N/DK/R
(AnDCause) If yes, What was the diagnosis? _______________
[Check box for Don't know/Refused]

Migrants from household

For the final section I would like to ask you about sources of income in your family.

(Contrib) How many adults contribute to the household income? ________
[Check box for Don't know/Refused]

(NonHHCont) Does everyone that contributes live **in** the household? Y/N/DK/R

(HHRemit) Does anyone that is part of your family send remittances to your household? Y/N/DK/R
(RemitRelat) If yes, what is that person's relationship to you? ____________________
[Check box for Don't know/Refused]

(RemitLoc) If Yes, Does the person send remittances from another parto f the country or from outside the country?
Inside the country
Another country: specify ______________________________
Don't know/Refused

(RemitAmt) How much remittance income you receive each month? ______________
[Check box for Don't know/Refused]

(RemitPct) How does the remittance compare to income from all other sources?
- A small portion (<10%)
- A modest portion (>10 and <25%)
- A substantial portion (>25% and <50%)
- A majority (>50%)
- Almost all of it (>90%)
- Don’t know/Refused

(RemitUses) What have you used remittance income for?
- Build a new house
- Remodel existing house – what did you change?
- Buy property
- Buy animals
- Build animal enclosure
- Healthcare
- Schooling
- Start/expand a business
- Other: Specify_______________________________________
- Don’t know
- Refused

(Comments) Do you have any other thoughts about the topics we have talked about that you would like to share with me?

____
____
____

Thank you for your time in answering these questions. The information provided will be very helpful as we try to better understand the concerns people have about the health of their families. Knowing more about people’s health concerns and the activities they take part in will help us address these health concerns and, we hope, reduce childhood diarrheal illness.

If you have time, I would be interested to accompany you to your farm to get to know the area a bit better. Would it be possible to go with you the next time you go? Agreed/Refused

(SHMeasD) Distance to farm measured by GPS: _________________

(SHMeasT) Time to arrive at farm: __________________

(SHAltChg) Altitude change to farm: __________________

Incentive

To thank you for your time, I would like to offer you a small token of appreciation. This book is designed to help farmers learn about animal care and treating animal diseases in the field. I hope it will be helpful for you.
