# Supplementary material for: An exploratory assessment of human and animal health concerns of smallholder farmers in rural communities of Chimborazo, Ecuador
Source: PeerJ. 2022 Jan 17;9:e12208. doi: 10.7717/peerj.12208 (PMC8772447; doi:10.7717/peerj.12208)
Supplement: Supplemental Information 2 [file peerj-10-12208-s002.pdf]

Encuesta: Evaluación de las preocupaciones de salud humana y animal en las comunidades rurales de Chimborazo, Ecuador

Demografía

Gracias por aceptar participar en este estudio. Me gustaría empezar por conocer un poco sobre usted y su familia.

(HeadofHH) ¿Es Ud. el jefe de la familia? S/N/NS/R

(AgeRpt) ¿Cuántos años tiene? ________

(RaceEth) ¿Qué raza o razas se considera? (Marque todas las que correspondan)

Mestiza
Indígena/nativa
Hispana/latina
Blanca
Negra/Afroecuatoriana
Otra: especifique ______________________________________
No sabe
Rehusó

(PmryLang) ¿Qué idioma habla principalmente en casa?

Español
Quichua
Tanto el español como el quichua
Otro: especifique ______________________________________

(Landowner) ¿Es Ud. el dueño de la tierra que utiliza para cultivar? S/N/NS/R

Conformación doméstica

(TotalHH) ¿Cuántas personas viven en su hogar, ya sea a tiempo completo o parcial? ________

[Cuadrito de tachar para opción de no saber/rehusar]

Me gustaría saber más sobre las personas que viven en su hogar. Por cada categoría de edad que mencione, por favor dígame cuántas personas en su hogar están en esa categoría de edad. También dígame qué tipo de trabajo hace cada persona.

**Categoría de edad**	**Número**	**Ocupaciones individuales**	**Categorías**
Adultos (> 18 años, < 65 años)<br>(HHcatAdult)		(Adult1Occ): _ _<br>(Adult2Occ): _ _<br>(Adult3Occ): _ _<br>(Adult4Occ): _ _<br>(Adult5Occ): _ _	Agricultura<br>Casero<br>Autónomos<br>Profesional<br>Deshabilitado<br>Desempleados<br>Retirado<br>Otro
Personas mayores (≥ 65 años)<br>(HHcatSenior)		(Senior1Occ): _ _<br>(Senior2Occ): _ _<br>(Senior3Occ): _ _	Agricultura<br>Casero<br>Autónomos<br>Profesional<br>Deshabilitado<br>Desempleados

			Retirado<br>Otro
< 5 años<br>(HHcatLT5)		(LTF1Occ): _ _<br>(LTF2Occ): _ _<br>(LTF3Occ): _ _<br>(LTF4Occ): _ _	Estudiante<br>Aún no en la escuela
5-12 años<br>(HHcat512)		(Tween1Occ): _ _ _ _<br>(Tween2Occ): _ _ _ _<br>(Tween3Occ): _ _ _ _<br>(Tween4Occ): _ _ _ _	Estudiante<br>No en la escuela<br>Trabajando<br>Estudiante + trabajo
13-17 años<br>(HHcat1317)		(Adol1Occ): _ _ _ _<br>(Adol2Occ): _ _ _ _<br>(Adol3Occ): _ _ _ _<br>(Adol4Occ): _ _ _ _	Estudiante<br>No en la escuela<br>Trabajando<br>Estudiante + trabajo

Indicadores de estatus socioeconómico

(RespEduc) ¿Cuál es el nivel más alto de la escuela que ha completado?

Ninguna educación formal
Algo de escuela primaria
Completó la escuela primaria
Algo de escuela secundaria
Completó la escuela secundaria
Algo de universidad
Graduó de la Universidad
Estudio de postgrado
Programa de comercio/vocación
Otro: especifique ______________________________________
Rehusó

(Homeowner) ¿Ud. es el dueño de la propiedad donde vive o la alquila?

Dueño
Inquilino
Otro: especifique ______________________________________
No sabe/Rehusó

Detalles de la casa

(HHAldea) ¿Cuál es el nombre de la zona donde se encuentra su casa? (aldea/recinto/pueblo)
____
[Cuadrito de tachar para opción de no saber/rehusar]

(HHBdrms) ¿Cuántas habitaciones hay en la casa en la que vive? ________
[Cuadrito de tachar para opción de no saber/rehusar]

(HHRunWtr) ¿La casa en la que vive tiene agua corriente interior? S/N/NS/R

(HHInBath) ¿La casa en la que vive tiene un baño interior? S/N/NS/R
(HHInBNum) En caso afirmativo, ¿Cuántos baños interiores tiene? ________
[Cuadrito de tachar para opción de rehusado]

(HHLatrine) ¿La casa en la que vive tiene una letrina afuera que se usa? S/N/NS/R

(HHWash) Cuando está en casa, ¿se lava las manos después de usar el baño/letrina?
S/N/NS/R

(HHWashLoc) En caso afirmativo, ¿Dónde se lava las manos después de usar la letrina? ________________________________

Detalles de la finca

(SHLoc) La casa en la que vive, ¿es parte de la propiedad que cultiva? S/N/NS/R

(SHAldea) ¿Cuál es el nombre de la zona donde se encuentra su finca? (aldea/recinto) ________________________________
[Cuadrito de tachar para opción de no saber/rehusar]

(SHSize) ¿Cuál es el tamaño de la propiedad que usted cultiva? ______________
[Cuadrito de tachar para opción de no saber/rehusar]
(Calcular la respuesta en hectáreas si se da en otra medida)

(SHDist) ¿A qué distancia está la propiedad donde cultiva de la casa en la que vive? __________
[Cuadrito de tachar para opción de no saber/rehusar]

(TimetoSH) ¿Cuánto tiempo le lleva llegar allí? (minutos) __________
[Cuadrito de tachar para opción de no saber/rehusar]

(SHTrvlType) ¿Cómo suele viajar a la finca?
Caminando
Caballo/burro
Auto/camioneta
Motocicleta/todo terreno
Otro: especifique ____________________________________
No sabe/Rehusó

Ahora me gustaría preguntarle sobre los animales que cría.

¿Qué tipo de animales tiene en su casa o en la propiedad en la que se encuentra su casa?

**Tipo**	**Número**
Perro (HHDog)	
Gato (HHCat)	
Pollo (HHChkn)	
Pato/ganso (HHDuck)	
Otras aves de corral (HHPltry)	
Cuy (HHCuy)	
Conejo (HHRabbit)	
Oveja (HHSheep)	
Cabra (HHGoat)	
Ganado (HHCattle)	
Burro (HHDonkey)	
Caballo (HHHorse)	
Llamingo (HHLlama)	
Alpaca/Otro Camélido (HHAlpaca)	
Otro (HHOthAnimal)	
Especificar (HHOthAnSp): _ _	

¿Qué tipo de animales tiene en su finca?

**Tipo**	**Número**
Perro (SHDog)	
Gato (SHCat)	

Pollo (SHChkn)	
Pato/ganso (SHDuck)	
Otras aves de corral (SHPltry)	
Cuy (SHCuy)	
Conejo (SHRabbit)	
Oveja (SHSheep)	
Cabra (SHGoat)	
Ganado (SHCattle)	
Burro (SHDonkey)	
Caballo (SHHorse)	
Llamingo (SHLlama)	
Alpaca/Otro Camélido (SHAlpaca)	
Otro (SHOthAnimal)	
Especificar (SHOthAnSp): _ _	

(SHIrrig) ¿Su finca tiene agua para riego? S/N
(SHIrrigHow) En caso afirmativo, ¿Cómo se recopila/distribuye?
Canal/acequia
Estanque/cisterna/barriles de recogida de agua de lluvia
Sistema municipal de agua
Manantial natural (ojo de agua)
Otro: especifique ______________________________________
No sabe/Rehusó

(SHRunWtr) ¿La finca tiene agua corriente? S/N/NS/R
(SHWtrTyp) En caso afirmativo, ¿Es entubado (municipal) o natural? Entubado/natural/DK/R

(SHInBath) ¿La finca tiene un baño interior? S/N/NS/R

(SHLatrine) ¿La finca tiene una letrina? S/N/NS/R

(SHHWash) Cuando está en la finca, ¿se lava las manos después de usar el baño/letrina? S/N/NS/R

(SHHWashLoc) En caso afirmativo, ¿Dónde se lava las manos después de usar la letrina?
____

(SHHmWst) ¿Qué hace con los desechos humanos en la finca?
Utiliza el rio/riachuelo
Los entierra
Los quema
Guarda en estanque de retención
Hace compost
Utiliza biodigestor
Se aplica directamente a los cultivos
Otro: especifique ____
No sabe/Rehusó

(SHHmWUse) [Si se aplica a los cultivos] ¿En qué tipo de cultivos los usa?
____

(SHAnEncl) [En caso afirmativo a los animales en la finca] ¿La finca tiene algún cerramiento de animales? S/N/NS/R
(SHEnclDes) En caso afirmativo, describa: ____________________________________

(SHAnWst) [En caso afirmativo a los animales en la finca] ¿Tiene la finca un sistema de manejo de desechos de animales de cualquier tipo? S/N/NS/R

(SHWstHow) En caso afirmativo, ¿de qué tipo?

Estanque de retención
Compost
Biodigestor
Otro: especifique ______________________________________
No sabe/Rehusó

(AnWstUse) [En caso afirmativo a los animales en la finca] ¿Para qué se usa los desechos de animales al final?

Se aplican sobre los cultivos
Compost
Los utiliza en el adobe
Los quema
Ningún uso/retención específico
Otro: especifique ______________________________________
No sabe/Rehusó

Problemas de salud y comportamientos

Ahora me gustaría preguntarle sobre algunas ideas que puede tener sobre la salud.

(PerHlth) ¿Cómo clasificaría su salud?

Excelente
Buena
Promedio (normal)
Pobre
Muy pobre
Rehusó

¿Cuáles son las tres cosas que más le preocupan de su salud?

(PerCrn1) ____
(PerCrn2) ____
(PerCrn3) ____

(StPerCrn) ¿Cómo clasificaría estos problemas de salud?
Son estos... (Seleccione todas las que correspondan)

¿Problemas de salud que tiene actualmente?
¿Problemas de salud que ha tenido en el pasado?
¿Problemas de salud que alguien en su familia tiene actualmente?
¿Problemas de salud que alguien en su familia ha tenido en el pasado?
¿Problemas de salud que alguien más que conozca tiene actualmente?
¿Problemas de salud que alguien más que conozca haya tenido en el pasado?
¿Problemas de salud que son comunes en la comunidad?
¿Problemas de salud de los que ha escuchado hablar?
(a través de amigos, conocidos, campañas publicitarias, proveedores médicos, otros)

¿Cuáles son las tres cosas que más le preocupan de la salud de su familia?

(FamCrn1) ____
(FamCrn2) ____
(FamCrn3) ____

(StFamCrn) ¿Cómo clasificaría estos problemas de salud?
Son estos... (Seleccione todas las que correspondan)
- ¿Problemas de salud que tiene actualmente?
- ¿Problemas de salud que ha tenido en el pasado?
- ¿Problemas de salud que alguien en su familia tiene actualmente?
- ¿Problemas de salud que alguien en su familia ha tenido en el pasado?
- ¿Problemas de salud que alguien más que conozca tiene actualmente?
- ¿Problemas de salud que alguien más que conozca haya tenido en el pasado?
- ¿Problemas de salud que son comunes en la comunidad?
- ¿Problemas de salud de los que ha escuchado hablar? (a través de amigos, conocidos, campañas publicitarias, proveedores médicos, otros)

¿Cuáles son las tres cosas que más le preocupan de la salud de sus animales?

(Anmlcrn1) ____
(Anmlcrn2) ____
(Anmlcrn3) ____

(StAnCrn) ¿Cómo clasificaría estos problemas de salud?
Son estos... (Seleccione todas las que correspondan)
- ¿Problemas de salud que sus animales tienen actualmente?
- ¿Problemas de salud que sus animales han tenido en el pasado?
- ¿Problemas de salud que tienen actualmente los animales de otra persona?
- ¿Problemas de salud que han tenido los animales de otra persona en el pasado?
- ¿Problemas de salud que son comunes en la comunidad?
- ¿Problemas de salud de los que ha escuchado hablar? (a través de amigos, conocidos, campañas publicitarias, veterinarios, otros)

Me gustaría hacerle algunas preguntas sobre algunas maneras en que usted y los niños de su hogar interactúan con sus animales.

(RawMAdlt) ¿Ud. toma leche sin pasteurizar? S/N/NS/R

(RawMPed) ¿Alguno de los niños en su hogar toma leche sin pasteurizar? S/N/NS/R

(RawWAdlt) ¿Ud. toma agua no tratada/sin hervir? S/N/NS/R

(RawWPed) ¿Alguno de los niños en su hogar toma agua no tratada/sin hervir? S/N/NS/R

(RecWAdlt) ¿Ud. se baña, juega o lava en el agua de lagos, ríos, riachuelos, canales u otras fuentes naturales? S/N/NS/R

(RecWPed) ¿Alguno de los niños en su hogar se baña, juega, o lava en el agua de lagos, ríos, riachuelos, canales, u otras fuentes naturales? S/N/NS/R

(BtchAdlt) ¿Ud. participa en la faena de animales? S/N/NS/R

(BtchPed) ¿Alguno de los niños en su hogar participa en la faena de animales? S/N/NS/R

(MilkAdlt) ¿Ud. ordeña animales? S/N/NS/R

(MilkPed) ¿Alguno de los niños en su hogar ordeña animales? S/N/NS/R

(AnWstAdlt) ¿Ud. interactúa con los desechos de animales? (limpieza del corral, compostaje, etc.) S/N/NS/R

(AnWstPed) ¿Alguno de los niños en su hogar interactúa con los desechos de animales? (limpieza de la pluma, compostaje, etc.) S/N/NS/R

(AdltDiar) En el último mes, ¿Ud. ha tenido algún episodio de diarrea? S/N/NS/R
(AdltDDays) en caso afirmativo, ¿Cuántos días duró la diarrea? __________
(AdltDCare) en caso afirmativo, ¿Buscó atención médica? S/N/NS/R
(AdltDDx) en caso afirmativo, ¿Recibió un diagnóstico para la causa de la diarrea? S/N/NS/R

(AdltDCause) en caso afirmativo, ¿Cuál fue el diagnóstico? _______________
[Cuadrito de tachar para opción de no saber/rehusar]

(PedDiar) En el último mes, ¿algún niño en el hogar ha tenido algún episodio de diarrea? S/N/NS/R
(PedDEpis) en caso afirmativo, ¿Cuántos de los niños? __________
[Si >3 tuvieran diarrea, anote los detalles de los 3 niños más pequeños]

(Ped1DAge) en caso afirmativo, ¿Cuál es la edad del niño/niña? __________
(Ped1DDay) en caso afirmativo, ¿Cuántos días duró la diarrea? __________
(Ped1DCare) en caso afirmativo, ¿Buscó atención médica para él/ella? S/N/NS/R
(Ped1DDx) en caso afirmativo, ¿Recibió un diagnóstico para la causa de la diarrea? S/N/NS/R

(Ped1DCse) en caso afirmativo, ¿Cuál fue el diagnóstico? ____________
[Cuadrito de tachar para opción de no saber/rehusar]

(Ped2DAge) en caso afirmativo, ¿Cuál es la edad del niño/niña? __________
(Ped2DDay) en caso afirmativo, ¿Cuántos días duró la diarrea? __________
(Ped2DCare) en caso afirmativo, ¿Buscó atención médica para él/ella? S/N/NS/R
(Ped2DDx) en caso afirmativo, ¿Recibió un diagnóstico para la causa de la diarrea? S/N/NS/R

(Ped2DCse) en caso afirmativo, ¿Cuál fue el diagnóstico? ____________
[Cuadrito de tachar para opción de no saber/rehusar]

(Ped3DAge) en caso afirmativo, ¿Cuál es la edad del niño/niña? __________
(Ped3DDay) en caso afirmativo, ¿Cuántos días duró la diarrea? __________
(Ped3DCare) en caso afirmativo, ¿Buscó atención médica para él/ella? S/N/NS/R
(Ped3DDx) en caso afirmativo, ¿Recibió un diagnóstico para la causa de la diarrea? S/N/NS/R

(Ped3DCse) en caso afirmativo, ¿Cuál fue el diagnóstico? ____________
[Cuadrito de tachar para opción de no saber/rehusar]

(AnDiar) En el último mes, ¿algún animal en el hogar o en su finca ha tenido algún episodio de diarrea? S/N/NS/R
(AnDDays) en caso afirmativo, ¿Cuántos días duró la diarrea? __________
(AnDCare) en caso afirmativo, ¿Buscó atención veterinaria para el animal? S/N/NS/R
(AnDDx) en caso afirmativo, ¿Recibió un diagnóstico para la cause de la diarrea? S/N/NS/R
(AnDCause) en caso afirmativo, ¿Cuál fue el diagnóstico? _______________
[Cuadrito de tachar para opción de no saber/rehusar]

Los emigrantes del hogar

Para la sección final me gustaría preguntarle acerca de las fuentes de ingreso en su familia.

(Contrib) ¿Cuántos adultos contribuyen al ingreso familiar? ________
[Cuadrito de tachar para opción de no saber/rehusar]

(NonHHCont) ¿Todos los que contribuyen viven en el hogar? S/N/NS/R

(HHRemit) ¿Alguien que es parte de su familia envía remesas a su hogar? S/N/NS/R
(RemitRelat) En caso afirmativo, ¿Cuál es la relación de esa persona con usted?
____
[Cuadrito de tachar para opción de no saber/rehusar]

(RemitLoc) En caso afirmativo, ¿Esa persona envía remesas desde otra parte del país o desde el exterior del país?
- Interior del país
- Otro país: especifique ________________________________
- No sabe/Rehusó

(RemitAmt) ¿Cuánto recibe de remesa cada mes? ______________
[Cuadrito de tachar para opción de no saber/rehusar]

(RemitPct) ¿Cómo se compara la remesa con los ingresos de todas las demás fuentes?
- Una pequeña porción (< 10%)
- Una porción modesta (> 10 y < 25%)
- Una porción sustancial (> 25% y < 50%)
- Más de la mitad (> 50%)
- Casi todo (> 90%)
- No sabe/Rehusó

(RemitUses) ¿Para qué ha utilizado las remesas?
- Construir una nueva casa
- Remodelación de la casa existente - ¿qué cambió?
- Comprar una propiedad
- Comprar animales
- Construir corral/cerramiento para animales
- Atención de salud
- Estudios
- Iniciar/expandir un negocio
- Otro: especifique _____________________________________
- No sabe
- Rehusó

(Comments) ¿Tiene alguna otra idea sobre los temas de que hemos hablado que le gustaría compartir conmigo?

____
____
____

Gracias por su tiempo para responder a estas preguntas. La información proporcionada será muy útil a medida que tratamos de comprender mejor las inquietudes que las personas tienen sobre la salud de sus familias. Saber más sobre las preocupaciones de salud de las personas y las actividades en las que participan nos ayudará a abordar estos problemas de salud y, esperamos, reducir las enfermedades diarreicas entre los niños.

Si tiene tiempo, me interesaría acompañarle a su finca para conocer el área un poco mejor. ¿Sería posible ir consigo la próxima vez que vaya? Asintió/Rehusó

(SHMeasD) Distancia a la finca medida por GPS: __________

(SHMeasT) Tiempo para llegar a la finca: __________

(SHAltChg) Cambio de altitud a la finca: __________

Incentivo
Para agradecerle por su tiempo, me gustaría ofrecerle una pequeña muestra de agradecimiento. Este libro está diseñado para ayudar a los agricultores a aprender sobre el cuidado de los animales y el tratamiento de enfermedades animales en el campo. Espero que le sea útil.
